# Structural basis for oxidative decarboxylation of lignin-derived aromatics by the fungal flavoprotein monooxygenase PcMNX1

**DOI:** 10.1016/j.jbc.2026.113411

**Published:** 2026-08-07

**Authors:** Reini Mori, Hiromitsu Suzuki, Takuya Ishida, Kiyota Sakai, Sora Yamaguchi, Naoki Sunagawa, Kiyohiko Igarashi, Masashi Kato, Motoyuki Shimizu

**Affiliations:** 1 Department of Applied Biological Chemistry, Faculty of Agriculture, Meijo University, Nagoya, Japan; 2 Graduate School of Agricultural and Life Sciences, The University of Tokyo, Tokyo, Japan

**Keywords:** lignin degradation, syringic acid, flavoprotein monooxygenase, oxidative decarboxylation, enzyme structure, *Phanerochaete chrysosporium*

## Abstract

Lignin depolymerization by white-rot fungi generates diverse aromatic compounds derived from hydroxyphenyl (H), guaiacyl (G), and syringyl (S) units. Although the metabolic pathways for G- and H-unit–derived aromatics have been studied, the enzymatic step responsible for the oxidative decarboxylation of the S-unit intermediate syringic acid (SA) has remained unknown. Here, we identify PcMNX1, a group A flavoprotein monooxygenase (FPMO) from the white-rot fungus *Phanerochaete chrysosporium*, as the enzyme catalyzing this missing step. Recombinant PcMNX1 catalyzed the NAD(P)H-dependent oxidative decarboxylation of SA to dimethoxyhydroquinone and also converted other lignin-derived aromatics, including vanillic acid and 4-hydroxybenzoic acid, with markedly higher catalytic efficiency than the closely related enzyme GsMNX1 from *Gelatoporia (Ceriporiopsis) subvermispora*. The crystal structure of PcMNX1 was determined at 2.00 Å resolution, revealing a typical group A FPMO fold with FAD bound in the “*out*” conformation. Structure-guided mutagenesis demonstrated that His247 functions as the catalytic base required for decarboxylative hydroxylation. Comparative structural analysis with bacterial 3-hydroxybenzoate 6-hydroxylase indicated that subtle substitutions in active-site residues alter substrate positioning and reaction outcomes. Consistent with this hypothesis, introduction of PcMNX1-type residues into 3-hydroxybenzoate 6-hydroxylase conferred decarboxylation activity toward lignin-derived aromatics. Furthermore, enlargement of the PcMNX1 active-site cavity through the L264A substitution markedly enhanced SA conversion. Together, these findings demonstrate that PcMNX1 catalyzes the oxidative decarboxylation of SA and reveal how subtle active-site remodeling diversifies the catalytic repertoire of closely related group A FPMOs involved in lignin-derived aromatic metabolism.

Lignin is a heterogeneous phenylpropanoid polymer and one of the most abundant biomaterials on Earth (1, 2). Its degradation plays a critical role in the global carbon cycle. White-rot basidiomycetes are capable of complete lignin mineralization and therefore represent key organisms in lignin turnover (2, 3, 4, 5). In these fungi, extracellular lignin peroxidases and manganese peroxidases constitute a unique enzymatic system for lignin conversion (6, 7). These enzymes were first isolated from *Phanerochaete chrysosporium*, a well-studied white-rot basidiomycete, and have since been identified in several other ligninolytic fungi (3, 4, 5). White-rot fungi initiate lignin depolymerization extracellularly using these nonspecific one-electron oxidizing enzymes, generating a variety of low-molecular-weight aromatic compounds derived from guaiacyl (G), syringyl (S), and hydroxyphenyl (H) units. Representative products include vanillin, vanillic acid (VA), syringaldehyde, SA, 4-hydroxybenzaldehyde, and 4-hydroxybenzoic acid (4-HBA) (4, 5, 6, 7), which are subsequently metabolized intracellularly. Among these intermediates, the S-unit–derived compound SA represents a key metabolite in lignin biodegradation by white-rot basidiomycetes (8). However, the metabolic pathway responsible for SA degradation remains largely unresolved in these fungi.

In contrast, the metabolism of G- and H-unit–derived lignin aromatics by white-rot basidiomycetes has been studied extensively (9, 10). A recent study by Kuatsjah *et al.* (10) provided biochemical and structural characterization of the complete hydroxyquinol pathway from 4-HBA to β-ketoadipate in *Trametes versicolor* and *Gelatoporia (Ceriporiopsis) subvermispora*. The G-unit compound vanillin and the H-unit compound 4-hydroxybenzaldehyde are oxidized to VA and 4-HBA, respectively (9, 10). These aromatic acids subsequently undergo oxidative decarboxylation to yield methoxyhydroquinone (MHQ) and hydroquinone (HQ), respectively, through 4-HBA 1-hydroxylase–type reactions catalyzed by flavoprotein monooxygenases (FPMOs) from white-rot fungi (9, 10). One such enzyme, GsMNX1 from *G.* *subvermispora* (JGI MycoCosm Protein ID: 120062), belongs to the group A FPMO family and catalyzes the oxidative decarboxylation of 4-HBA, protocatechuic acid (PCA), and VA. Although it exhibits the highest catalytic efficiency toward PCA, it shows no detectable activity toward SA (10). The oxidative degradation pathway of 4-HBA was first reported in the ascomycetous yeast *Candida parapsilosis*, and the responsible FPMOs were initially characterized by Eppink *et al*. (11). Notably, these enzymes preferentially act on G- and H-unit–derived compounds, and oxidative decarboxylation of SA has not been reported. The metabolic entry point for the S-unit–derived compound SA in white-rot fungi therefore remains unresolved, highlighting a critical gap in our understanding of lignin-derived aromatic catabolism.

More than 3 decades ago, studies demonstrated that MHQ in white-rot fungi can be demethoxylated to 1,2,4-trihydroxybenzene (THB), which subsequently undergoes aromatic ring cleavage by THB dioxygenases, including intradiol dioxygenase 1 (IDD1) and intradiol dioxygenase 2 (IDD2) from *P. chrysosporium* (12, 13). In addition, we previously showed that IDD2 catalyzes the ring cleavage of 6-methoxy-1,2,4-trihydroxybenzene (6-MTHB) (13). Despite these advances, the enzymes responsible for the demethoxylation of G- and S-unit-derived lignin fragments in white-rot basidiomycetes remain unidentified (10). In contrast, a recent study demonstrated that extracellular short-chain polyphenol oxidases from lignocellulose-degrading ascomycetes catalyze the *ortho*-hydroxylation and oxidative demethoxylation of G- and S-type phenolics, producing methoxy-*ortho*-diphenols (14). Whether analogous enzymatic systems exist in white-rot basidiomycetes remains unclear. Recently, we biochemically characterized two homogentisate dioxygenase homologs, methoxyhydroquinone dioxygenase 1 (MHQD1) and MHQD2, from the white-rot fungus *P. chrysosporium* (15). Both enzymes catalyze the ring cleavage of MHQ and 2,6-dimethoxyhydroquinone (DMHQ) (15). However, the enzyme(s) responsible for the oxidative decarboxylation of SA to DMHQ—an essential step linking S-unit-derived lignin fragments to downstream ring-cleavage reactions—have not yet been identified in white-rot fungi.

In the present study, we sought to identify the FPMO in *P. chrysosporium* capable of catalyzing the oxidative decarboxylation of SA. We characterized the biochemical and structural properties of PcMNX1 and discuss its potential role in the metabolic pathway for S-unit-derived lignin fragments in *P. chrysosporium*.

## Results

### Conversion of SA by cell-free extracts of *P. chrysosporium*

**Figure 1:**
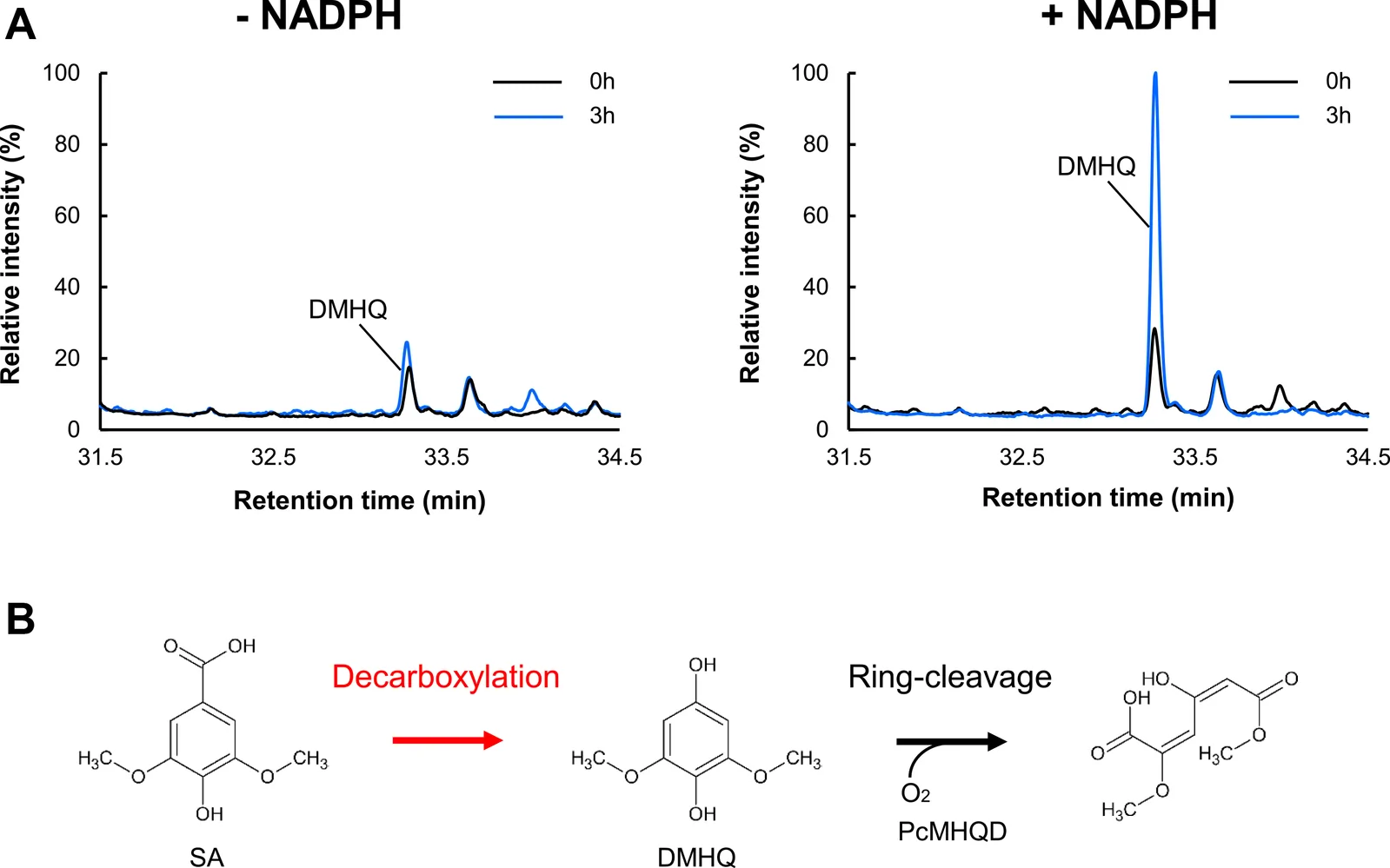
**Conversion of syringic acid by cell-free extracts of *Phanerochaete chrysosporium*.***A*, cell-free extracts of *P. chrysosporium* were incubated with 2 mM SA, 4 mM NADPH, and 1 mM DTT in 20 mM Hepes buffer (pH 7.5) at 20 °C for 3 h. Reaction products were analyzed by GC–MS. The *black trace* represents the chromatogram at 0 h, and the *blue trace* represents the chromatogram after 3 h of incubation. Experiments were performed in triplicate, and representative chromatograms are shown. *B*, proposed pathway for SA conversion in *P. chrysosporium*. *Red arrows* indicate predicted reactions, whereas *black arrows* indicate experimentally validated reactions catalyzed by PcMHQD.

In our previous study, we demonstrated that *P. chrysosporium* converts SA into DMHQ, 5-hydroxyvanillic acid (5-HVA), and 6-MTHB through decarboxylation and demethylation reactions (13). To further investigate the initial step of SA metabolism, cell-free extracts prepared from *P. chrysosporium* were incubated with SA in the presence or absence of NADPH. Under these conditions, DMHQ production increased markedly in an NADPH-dependent manner, whereas 5-HVA and 6-MTHB were not detected (Fig. 1*A*). These results indicate the presence of an NADPH-dependent enzyme that catalyzes the initial decarboxylation step of SA metabolism in *P. chrysosporium*. Furthermore, the data suggest that oxidative decarboxylation followed by aromatic ring cleavage is likely to represent a major metabolic route for SA in this fungus (Fig. 1*B*).

### Identification of candidates for syringate 1-hydroxylases in the *P. chrysosporium* genome

As shown in Figure 1, SA in *P. chrysosporium* is initially subjected to NADPH-dependent decarboxylation. In contrast, previously characterized group A FPMOs, including GsMNX1 and CpMNX1, catalyze the oxidative decarboxylation of VA and 4-HBA, but exhibit no detectable activity toward SA (10, 11). Based on these observations, we hypothesized that a distinct FPMO homolog sharing sequence similarity with GsMNX1 might catalyze the oxidative decarboxylation of SA. The genome of *P. chrysosporium* contains 57 predicted FPMO genes, comprising 39 group A enzymes and 18 group B enzymes (Fig. S1*A*) (16). To identify candidate enzymes responsible for SA 1-hydroxylation accompanied by decarboxylation, the amino acid sequence of GsMNX1 (JGI MycoCosm Protein ID: 120062) was used as a query in BLAST searches against the *P. chrysosporium* genome database (https://mycocosm.jgi.doe.gov/Phchr4_2/Phchr4_2.home.html). This analysis revealed that GsMNX1 shares 34.8%, 37.0%, 37.4%, 35.8%, and 69.0% amino acid sequence identity with PcFPMO4 (JGI MycoCosm Protein ID: 6222556), PcFPMO5 (6342839), PcFPMO6 (6212972), PcFPMO7 (6212917), and PcFPMO8 (6247437), respectively. To further evaluate their evolutionary relationships, phylogenetic analysis of PcFPMOs together with biochemically characterized group A FPMOs was performed using the neighbor-joining method with 1000 bootstrap replicates. PcFPMO4–8 clustered within the same phylogenetic branch as GsMNX1 and CpMNX1 (Fig. S1*B*). In addition, multiple sequence alignment of PcFPMO4–8 with characterized FPMOs revealed strong conservation of residues implicated in general base catalysis and substrate binding, particularly among PcFPMO8, GsMNX1, and CpMNX1 (Fig. S2). Based on these sequence and phylogenetic analyses, the five PcFPMO candidates were selected for heterologous expression in *Escherichia coli* for functional characterization.

### Catalytic properties of PcFPMOs

Five PcFPMO candidates were heterologously expressed using the *E. coli* pQE60 expression system. Among them, C-terminally His_6_-tagged PcFPMO5, PcFPMO7, and PcFPMO8 were obtained as soluble proteins (Fig. S3*A*), whereas PcFPMO4 and PcFPMO6 were expressed as inclusion bodies (data not shown). The soluble recombinant proteins were purified by nickel-affinity chromatography. The UV–visible absorption spectra of the purified enzymes exhibited characteristic flavoprotein features, with absorption maxima at approximately 380 and 450 nm (Fig. S3*B*), consistent with the presence of FAD.

**Figure 2:**
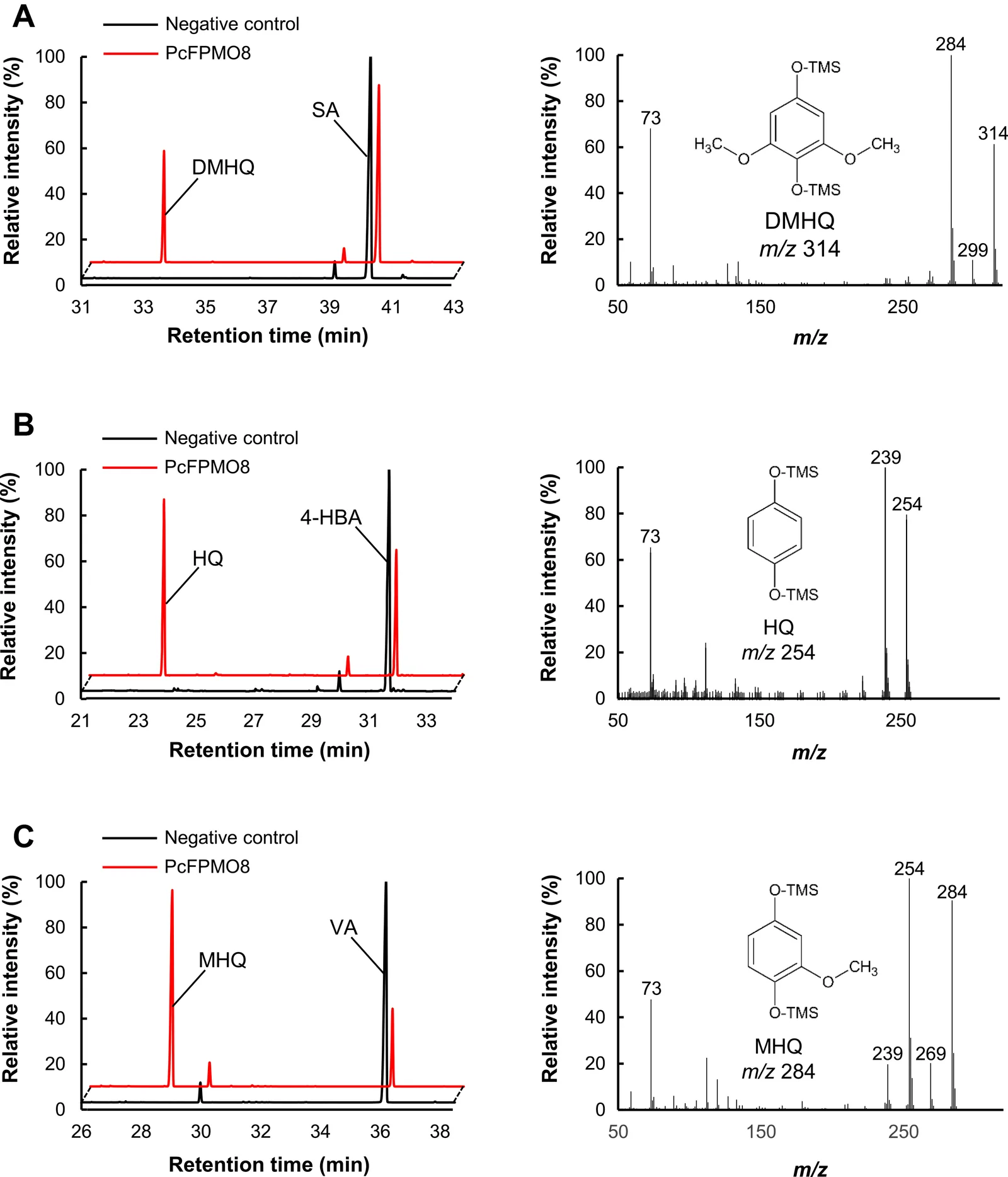
**GC–MS analysis of reaction products generated by PcMNX1.** Reaction products generated from SA (*A*), 4-HBA (*B*), and VA (*C*) were analyzed by GC–MS after TMS derivatization. Mass spectra of the reaction products—DMHQ (*A*), HQ (*B*), and MHQ (*C*)—were obtained from GC peaks with retention times of 33.4 min (*A*), 23.9 min (*B*), and 28.7 min (*C*). The *black trace* represents the reaction without enzyme, and the *red trace* represents the reaction in the presence of PcMNX1. Experiments were performed in triplicate, and representative chromatograms are shown. 4-HBA, 4-hydroxybenzoic acid; DMHQ, dimethoxyhydroquinone; HQ, hydroquinone; MHQ, methoxyhydroquinone; SA, syringic acid; TMS, trimethylsilyl; VA, vanillic acid.

To evaluate catalytic activity, recombinant PcFPMO5, PcFPMO7, and PcFPMO8 were incubated with SA using NADPH or NADH as electron donors, and the reaction products were analyzed by GC–MS. The decarboxylated product was identified as DMHQ based on the mass spectrum of its trimethylsilyl derivative (Figs. 2*A* and S4). Among the three enzymes tested, only PcFPMO8 catalyzed the oxidative decarboxylation of SA to DMHQ. PcFPMO8 utilized both NADPH and NADH as cofactors, exhibiting higher catalytic efficiency with NADH (Table 1). To our knowledge, this study represents the first report describing syringate 1-hydroxylase activity within the FPMO superfamily. Under the same experimental conditions, PcFPMO8—hereafter referred to as PcMNX1—also catalyzed the oxidative decarboxylation of 4-HBA and VA, producing HQ and MHQ, respectively (Fig. 2, *B* and *C*). In contrast, PcFPMO5 and PcFPMO7 showed no detectable activity toward these substrates (Fig. S4, *A*–*C*). We next examined additional aromatic substrates. PcMNX1 converted PCA, gallic acid (GA), and 5-HVA to their respective decarboxylated products—THB, 1,3,4,5-tetrahydroxybenzene (TetraHB), and 6-MTHB, respectively— as confirmed by GC–MS analysis (Fig. S5, *A*–*C*). Furthermore, positional isomers of 4-HBA were examined as substrates. PcMNX1 catalyzed the hydroxylation of 3-HBA to yield 2,5-dihydroxybenzoic acid (2,5-DHB) (Fig. S5*D*), whereas no detectable activity was observed toward 2-hydroxybenzoic acid (data not shown). The effects of pH and temperature on PcMNX1 activity were subsequently examined using VA as the substrate. The enzyme exhibited optimal activity at pH 8.0 (Fig. S6*A*) and 20 °C (Fig. S6*B*).

**Table 1:** Kinetic parameters and hydroxylation efficiencies of PcMNX1

Substrate	K_m_ (μM)	k_cat_ (s^−1^)	k_cat_/K_m_ (s^−1^M^−1^)	Hydroxylation efficiency (%)
4-Hydroxybenzoic acid	19 ± 0.6	20.0 ± 0.7	1.0 × 10^6^	76 ± 1.4
Vanillic acid	8 ± 0.4	17.8 ± 0.5	2.4 × 10^6^	59 ± 0.4
Syringic acid	518 ± 15.4	3.7 ± 0.1	7.0 × 10^3^	33 ± 2.5
Protocatechuic acid	62 ± 7.4	8.5 ± 0.5	1.3 × 10^5^	56 ± 1.6
Gallic acid	265 ± 3.4	3.2 ± 0.1	1.2 × 10^4^	67 ± 0.9
5-Hydroxyvanillic acid	474 ± 20.4	6.0 ± 0.1	1.3 × 10^4^	85 ± 3.0
3-Hydroxybenzoic acid	63 ± 3.4	1.3 ± 0.1	2.1 × 10^4^	59 ± 8.5
NADPH	30 ± 0.9	14.8 ± 0.3	5.0 × 10^5^	–
NADH	9 ± 0.2	14.5 ± 0.2	1.6 × 10^6^	–

Kinetic parameters and hydroxylation efficiencies were determined in 0.5 ml reaction mixtures containing PcMNX1, 200 μM NAD(P)H, and 5 μl of substrate solution (0–500 mM in dimethyl sulfoxide) in 20 mM Hepes buffer (pH 8.0). A PcMNX1 concentration of 5 nM was used for kinetic analyses with NAD(P)H, 4-HBA, VA, and PCA, whereas a concentration of 0.1 μM was used for analyses with SA, GA, 5-HVA, and 3-HBA. Reactions were initiated by the addition of NAD(P)H and incubated at 20 °C. The decrease in absorbance at 340 nm was monitored spectrophotometrically, and substrate hydroxylation was quantified by HPLC. The kinetic parameters *K*_m_ and *k*_cat_ were calculated by fitting the initial reaction rates to the Michaelis–Menten equation. Data represent the mean ± SD from three independent experiments.

### Hydroxylation efficiencies and kinetic parameters

Hydroxylation efficiency is an important indicator of catalytic coupling in FPMOs, reflecting how effectively reducing equivalents derived from NAD(P)H are utilized for productive substrate hydroxylation rather than uncoupled oxygen reduction (17). Based on this concept, the hydroxylation efficiencies of PcMNX1 were determined by monitoring NADH oxidation at 340 nm and quantifying product formation by HPLC. PcMNX1 exhibited the highest hydroxylation efficiency toward 5-HVA (85%), followed by 4-HBA (76%), GA (67%), VA (59%), 3-HBA (59%), PCA (56%), and SA (33%) (Table 1). The steady-state kinetic parameters of PcMNX1 were also determined (Table 1). Among the seven substrates tested, VA exhibited the highest catalytic efficiency (*k*_cat_/*K*_m_ = 2.4 × 10^6^ s^−1^M^−1^), consistent with its low *K*_m_ value (8 μM). In contrast, 4-HBA displayed the highest turnover number (*k*_cat_ = 20.0 s^−1^) but a lower catalytic efficiency than VA because of its higher *K*_m_ value (19 μM). SA showed markedly lower catalytic efficiency (*k*_cat_/*K*_m_ = 7.0 × 10^3^ s^−1^M^−1^), primarily due to its high *K*_m_ value (518 μM). Consistent with its cofactor preference, PcMNX1 utilized both NADPH and NADH, exhibiting higher catalytic efficiency with NADH (Table 1). The catalytic efficiencies of PcMNX1 toward VA and 4-HBA were approximately 48-fold and 9-fold higher, respectively, than the corresponding values reported for GsMNX1 (10), despite the 69% amino acid sequence identity between the two enzymes. Furthermore, whereas GsMNX1 exhibits a strong preference for NADPH over NADH (10), PcMNX1 showed higher catalytic efficiency with NADH, revealing a marked difference in cofactor preference between these closely related enzymes.

### Overall structure of PcMNX1

**Figure 3:**
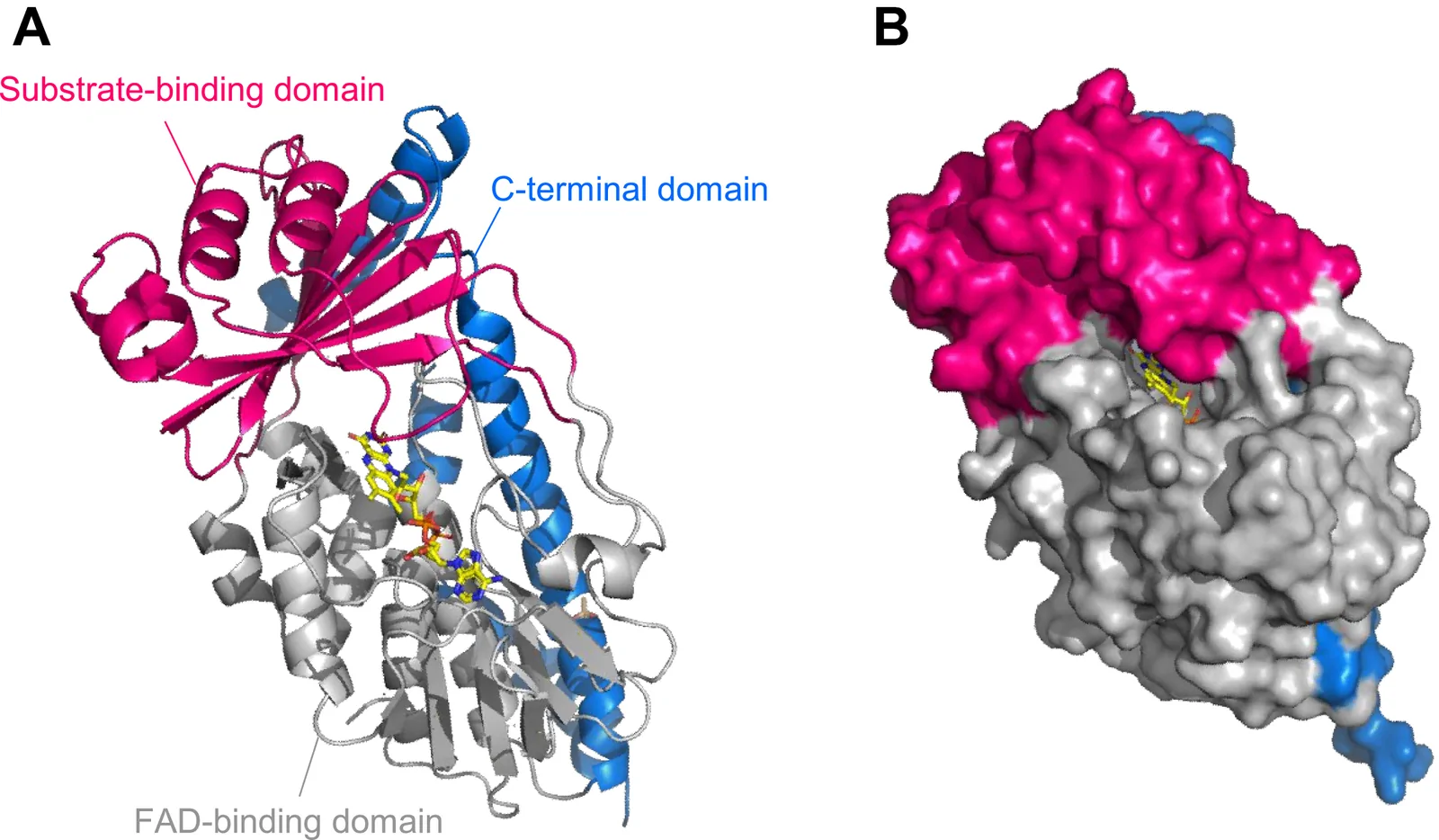
**Overall structure of PcMNX1.***A*, ribbon representation of PcMNX1. The FAD-binding, substrate-binding, and C-terminal domains are shown in *gray*, *magenta*, and *blue*, respectively. The bound FAD cofactor is shown as *yellow sticks*. *B*, surface representation of PcMNX1 highlighting the domain organization. FAD, flavin adenine dinucleotide.

To investigate the structural basis of the catalytic mechanism, the crystal structure of PcMNX1 was determined using diffraction data collected to 2.00 Å resolution (PDB ID: 8X38; Fig. 3 and Table S1). The final structural model contains one molecule of FAD and one acetate ion. All residues were well defined in the electron density map except for the N-terminal 25 residues (M1–V25) and the C-terminal 21 residues, which include a spacer sequence and the C-terminal His_6_ tag. Similar to other group A FPMOs, PcMNX1 adopts a three-domain architecture (Fig. 3) and shows structural similarity to GsMNX1, 3-hydroxybenzoate 6-hydroxylase (3HB6H) from *Rhodococcus jostii* RHA1 (PDB ID: 4BK1; 39.1% sequence identity), and 6-hydroxynicotinic acid 3-hydroxylase (NicC) from *Pseudomonas putida* KT2440 (PDB ID: 5EOW; 48.5% sequence identity) (10, 18, 19). The asymmetric unit contains a single PcMNX1 molecule, consistent with the apparent molecular mass determined by size-exclusion chromatography (∼51 kDa), indicating that PcMNX1 exists as a monomer in solution.

A single FAD molecule is bound within a cavity approximately 25 Å long and 8 Å wide in the PcMNX1 monomer. The cofactor adopts the “*out*” conformation (Fig. 3). The FAD-binding domain contains a characteristic Rossmann fold and conserved FAD-binding motifs, including the GXGXXG motif (G43–G48) and the DG motif (D189–G190), which interact with the ADP moiety of the cofactor (20). The ADP and ribityl moieties of FAD are tightly anchored within the FAD-binding domain through numerous direct and water-mediated hydrogen-bonds (Fig. S7*A*). The isoalloxazine ring of FAD is positioned within a hydrophobic pocket (Fig. S7*A*) and is stabilized by parallel π–π stacking interactions with the side chain of Trp274. This arrangement resembles that observed in other group A FPMOs adopting the “*out*” conformation in the ligand-free state (21) (Fig. S7*B*).

### Structural basis of substrate recognition in PcMNX1

**Figure 4:**
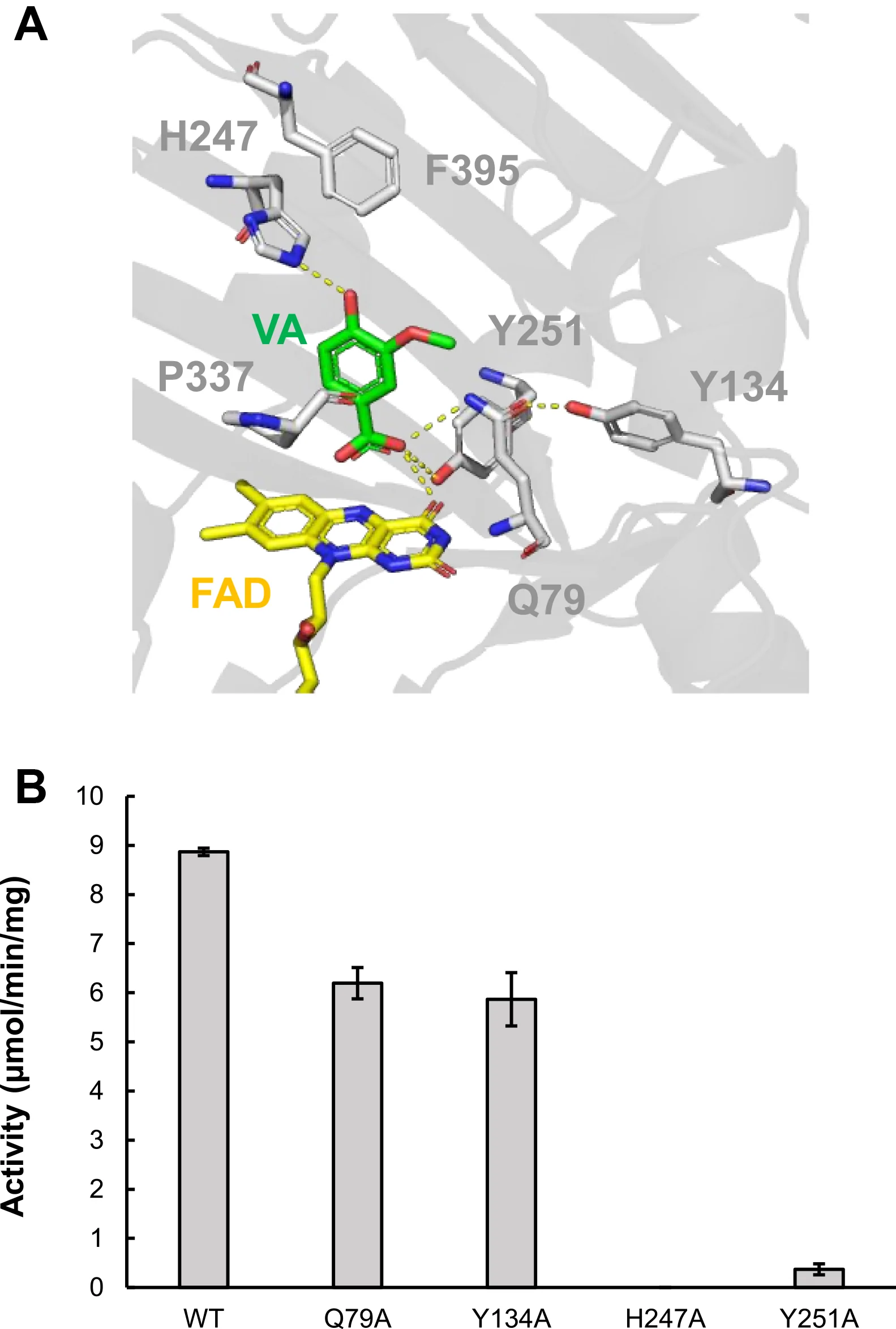
**Putative active-site pocket of PcMNX1.***A*, predicted active-site model of PcMNX1 with VA docked into the catalytic pocket using the MOE program. Carbon atoms of PcMNX1, VA, and FAD are shown in *gray*, *green*, and *yellow*, respectively; nitrogen and oxygen atoms are shown in *blue* and *red*, respectively. Potential hydrogen-bond interactions between the enzyme and VA are indicated by *yellow dashed lines*. *B*, specific activities of PcMNX1 variants toward VA. Reactions were performed in 0.5-mL mixtures containing 5 nM enzyme, 200 μM NAD(P)H, and 200 μM VA in 20 mM Hepes buffer (pH 8.0) at 20 °C for 10 min. Reaction products were quantified by HPLC. Activities are expressed as μmol min^−1^ mg^−1^ protein. Data represent the mean ± S.D. from three independent experiments. FAD, flavin adenine dinucleotide; MOE, Molecular Operating Environment; VA, vanillic acid.

Attempts to crystallize PcMNX1 in complex with VA were unsuccessful. Therefore, the putative VA-binding site was investigated using a structure-based computational approach. Group A FPMOs are known to undergo conformational transitions of the FAD cofactor between open and closed states during catalysis, enabling electron transfer from reduced NAD(P)H, followed by oxygen activation and substrate hydroxylation (17, 22, 23). Because FAD in the PcMNX1 crystal structure adopts the “*out*” conformation (Fig. 3), a structural model of PcMNX1 with FAD in the catalytically relevant “*in*” conformation was generated using AlphaFold3 (Fig. S8, *A* and *B*). The rationale for this approach is that the PcMNX1 crystal structure captures FAD in the catalytically inactive “*out*” conformation, thereby precluding its direct use as a docking template. The validity of the AlphaFold3-derived “*in*” model is supported by its close agreement with the experimentally determined “*in*” conformation of GsMNX1 (PDB ID: 8R2T) (10), which superimposes onto the PcMNX1 model with an RMSD of 0.8 Å over 357 Cα atoms. Using this modeled structure, VA was docked into the putative active-site cavity using the Molecular Operating Environment program. The predicted binding mode was further supported by structural superposition with GsMNX1 and 3HB6H (10, 18). As shown in Figure 4*A*, VA is positioned within the active site through four hydrogen bonds that orient the substrate for decarboxylative hydroxylation at the C1 position. Specifically, the 4-hydroxyl group of VA forms a hydrogen bond with His247, whereas the 1-carboxylate group interacts with Gln79, Tyr251, and the isoalloxazine ring of FAD. Based on these structural observations, we hypothesized that Gln79, His247, and Tyr251 play critical roles in substrate binding and catalysis. To test this hypothesis, variants containing substitutions at the predicted active-site residues (Q79A, Y134A, H247A, and Y251A) were generated and their catalytic activities toward VA were evaluated (Fig. 4*B*). The H247A variant was completely inactive, indicating that His247 functions as the general base catalyst in PcMNX1. Substitution of Gln79 resulted in a moderate reduction in catalytic activity, consistent with its proposed role in substrate anchoring and orientation. In contrast, the Y251A variant exhibited a pronounced loss of activity, suggesting that the aromatic side chain of Tyr251 is critical for proper substrate positioning, while its hydroxyl group contributes to optimal substrate binding. Similar to the catalytic architecture observed in 3HB6H, Tyr134 forms a hydrogen bond with Gln79, thereby stabilizing the orientation of the Gln side chain and promoting its interaction with the substrate carboxylate (18). Consistent with this structural role, the Y134A variant displayed moderately reduced activity relative to the WT enzyme. These results collectively support the importance of a hydrogen-bonding network that stabilizes the productive substrate-binding conformation. Taken together, these findings support a reaction mechanism in which VA, activated by His247, undergoes *ipso* attack by the flavin C4a-hydroperoxide intermediate, leading to decarboxylative hydroxylation (Fig. S9).

To further investigate the functional divergence within the PcFPMO family, three-dimensional structural models of PcFPMO4–7 were generated using AlphaFold3, and their putative substrate-binding sites were analyzed (Fig. S10). Although PcFPMO4–7 belong to the same phylogenetic cluster as PcFPMO8 (PcMNX1), clear differences were observed in key catalytic residues. In PcFPMO4–7, the residue corresponding to His247, the putative general base catalyst of PcMNX1, is replaced by nonbasic amino acids: Gly230 in PcFPMO4, Cys232 in PcFPMO5, Ser226 in PcFPMO6, and Ser227 in PcFPMO7 (Fig. S10). Activation of the phenolic hydroxyl group may occur through alternative mechanisms, such as interactions with other amino acid residues or a hydrogen-bonding water network, and may not necessarily require direct deprotonation. Phenolic hydroxyl activation *via* a water network has been reported for a group A FPMO (24). Nevertheless, PcFPMO5 and PcFPMO7 exhibited no detectable catalytic activity toward 4-HBA, VA, or SA (Fig. S4). These observations suggest that substitution of the putative catalytic histidine may be one factor contributing to the lack of detectable oxidative decarboxylation activity in these enzymes.

### Comparative analysis of PcMNX1 and GsMNX1

GsMNX1 shares 69.0% amino acid sequence identity with PcMNX1 and exhibits high structural similarity. Despite this close similarity, only PcMNX1 catalyzes the decarboxylation of SA, whereas GsMNX1 shows no detectable activity toward this substrate (10). To elucidate the structural basis underlying this difference in substrate specificity, the active-site pockets of PcMNX1 and GsMNX1 were compared using SA-docked structural models (Fig. S11, *A* and *B*). Although the general base catalyst and key substrate-interacting residues are conserved between the two enzymes, the size and geometry of the active-site cavities differ substantially. The active-site cavity of PcMNX1 (Fig. S11*A*) is larger than that of GsMNX1 (Fig. S11*B*), suggesting that steric constraints influence SA recognition. In PcMNX1, the smaller Ile107 residue creates sufficient space to accommodate the C5 methoxy group of SA, thereby permitting productive substrate binding and catalysis. In contrast, the corresponding residue in GsMNX1, Met93, is bulkier and reduces the available cavity volume, likely preventing proper accommodation of SA. To test this structural hypothesis, an I107M variant of PcMNX1 was generated to mimic the GsMNX1-type active site. The PcMNX1_I107M variant completely lost decarboxylative activity toward SA (Fig. S11*C* and Table 2) while retaining catalytic activity toward VA (Fig. S11*D* and Table 2). These results indicate that Ile107 is a key determinant controlling active-site cavity size and SA specificity in PcMNX1.

**Table 2:** Kinetic parameters of PcMNX1 variants

Substrate	Variant	K_m_ (μM)	k_cat_ (s^−1^)	k_cat_/K_m_ (s^−1^M^−1^)	Hydroxylation efficiency (%)
Vanillic acid	I107M	275 ± 13.0	7.8 ± 0.1	2.8 × 10^4^	78 ± 0.3
	L264A	98 ± 5.1	13 ± 0.6	1.3 × 10^5^	82 ± 1.1
Syringic acid	I107M	N.D.	N.D.	N.D.	–
	L264A	191 ± 15.4	25 ± 1.4	1.3 × 10^5^	33 ± 0.4

Kinetic parameters for PcMNX1 variants were determined under the same experimental conditions described in Table 1. Kinetic analyses of the PcMNX1 I107M and L264A variants were performed using enzyme concentrations of 0.1 μM and 20 nM, respectively. Data represent the mean ± SD from three independent experiments. N.D., not detected.

The ability of PcMNX1 to catalyze SA conversion therefore appears to depend on the dimensions of the active-site cavity. In PcMNX1, Leu264 forms part of the active-site cavity wall that accommodates the C3 methoxy group of SA (Fig. S12*A*). We hypothesized that substituting Leu264 with alanine would enlarge the available space within the cavity and thereby enhance catalytic activity toward SA (Fig. S12*B*). To test this hypothesis, an L264A variant of PcMNX1 was generated and biochemically characterized. The PcMNX1_L264A variant exhibited an approximately 18-fold increase in activity toward SA compared with the WT enzyme (Fig. S12*C* and Table 2), whereas its activity toward VA was slightly reduced (Fig. S12*D* and Table 2). These findings further support the conclusion that active-site cavity size is a tunable structural determinant governing SA reactivity.

### Bioconversion of SA by resting cells

To evaluate the biotechnological potential of the PcMNX1_L264A variant, resting cells expressing either WT PcMNX1 or the PcMNX1_L264A variant were prepared and subjected to SA bioconversion assays (Fig. S13*A*). In resting cells expressing WT PcMNX1, the SA concentration gradually decreased from 0.5 mM to approximately 0.38 mM after 60 min of incubation (Fig. S13*B*). SDS–PAGE analysis confirmed that the expression levels of WT PcMNX1 and the PcMNX1_L264A variant were comparable under these conditions (Fig. S13*A*). In contrast, resting cells expressing the PcMNX1_L264A variant exhibited a markedly accelerated decline in SA levels, with complete depletion observed within 60 min (Fig. S13*B*). Consistent with this enhanced substrate conversion, approximately 0.10 mM DMHQ, the decarboxylated product of SA, accumulated in cells expressing WT PcMNX1, whereas 0.38 mM DMHQ was produced in cells expressing the L264A variant (Fig. S13*B*). No detectable SA conversion was observed in resting cells harboring the empty pQE60 vector. Collectively, these results demonstrate that the L264A substitution substantially enhances SA bioconversion efficiency in the resting-cell system.

### Structural basis for the functional divergence between PcMNX1 and 3HB6H

**Figure 5:**
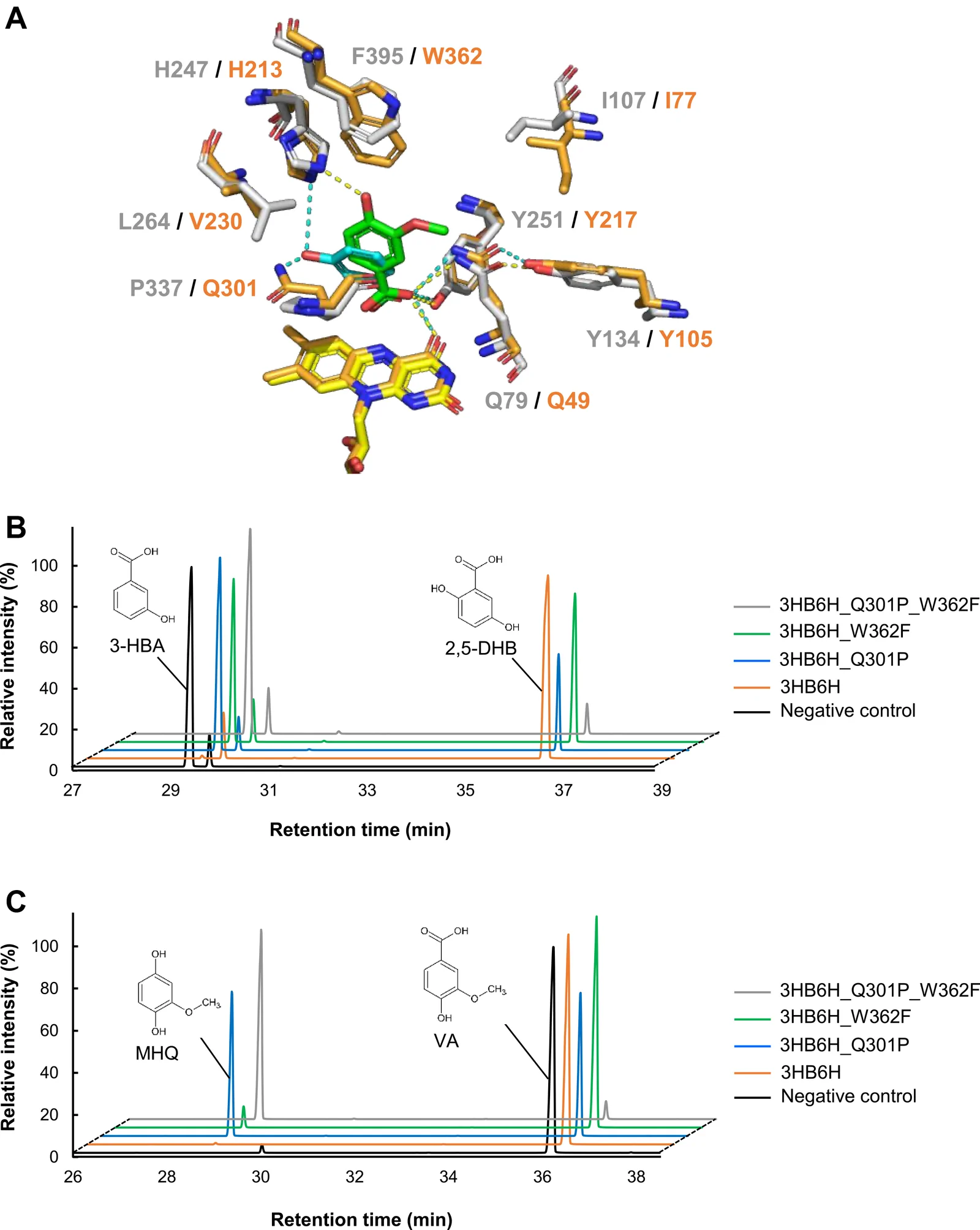
**Comparison of the active-site architectures of PcMNX1 and 3-hydroxybenzoate 6-hydroxylase.***A*, superposition of the active sites of PcMNX1 (*gray*) docked with VA (*green*) and 3HB6H from *Rhodococcus jostii* RHA1 bound to 3-HBA (*cyan*) (*orange*; PDB ID: 4BK1). The latter structure was solved as the H213S mutant; therefore, PDB ID 4BJZ was used to visualize His213. Predicted PcMNX1–VA interactions are shown as *yellow dashed lines*, whereas 3HB6H–3-HBA interactions are shown as *cyan dashed lines*. *B* and *C*, GC–MS analysis of reaction products generated from 3-HBA (*B*) and VA (*C*) following TMS derivatization. The reaction products 2,5-DHB (*B*) and MHQ (*C*) were detected at retention times of 36.5 min and 28.7 min, respectively. Chromatograms corresponding to reactions without enzyme, wild-type 3HB6H, the Q301P variant, the W362F variant, and the Q301P/W362F double variant are shown in *black*, *orange*, *blue*, *green*, and *gray*, respectively. Experiments were performed in triplicate, and representative chromatograms are shown. 2,5-DHB, 2,5-dihydroxybenzoic acid; 3HB6H, 3-hydroxybenzoate 6-hydroxylase; 3-HBA, 3-hydroxybenzoic acid; MHQ, methoxyhydroquinone.

PcMNX1 catalyzes both the decarboxylation of lignin-derived aromatic compounds and the hydroxylation of 3-HBA to yield 2,5-DHB (Fig. S5*D* and Table 1). In contrast, 3HB6H catalyzes the hydroxylation of 3-HBA and related derivatives but does not mediate decarboxylation reactions (24, 25). To elucidate the structural basis underlying this functional divergence, we performed comparative analyses of their catalytic activities and active-site architectures. The general base catalyst and key substrate-binding residues are conserved between PcMNX1 and 3HB6H (Fig. S2). However, distinct differences are observed in residues that contribute to substrate positioning. In 3HB6H, in addition to the catalytic residue His213, Gln301 forms a hydrogen bond with the hydroxyl group of 3-HBA (18, 24). In PcMNX1, the corresponding position is occupied by Pro337, which lacks the ability to form a comparable hydrogen bond with the hydroxyl group of VA (Fig. 5*A*). The interaction between Gln301 and 3-HBA in 3HB6H likely stabilizes a defined substrate orientation within the active-site pocket, positioning the flavin C4a-hydroperoxide intermediate proximal to the C6 position of the aromatic ring and thereby favoring hydroxylation over decarboxylation (Fig. 5*A*) (18, 24). In contrast, the absence of this hydrogen-bonding interaction in PcMNX1 may permit alternative substrate positioning, thereby enabling decarboxylative reactions. Additional differences are observed in residues adjacent to the catalytic center. In PcMNX1, Phe395 corresponds to Trp362 in 3HB6H. The larger side chain of Trp362 is expected to impose greater steric constraints on the substrate-binding pocket, thereby contributing to differences in substrate specificity (Fig. S14, *A* and *B*).

Based on these observations, we hypothesized that Gln301 and Trp362 of 3HB6H, corresponding to Pro337 and Phe395 of PcMNX1, play key roles in determining the size and geometry of the active-site cavity. To test this hypothesis, Q301P, W362F, and Q301P/W362F variants of 3HB6H were generated, and their catalytic activities toward 3-HBA and VA were determined (Fig. 5, *B* and *C*). WT 3HB6H and the W362F variant efficiently converted 3-HBA to 2,5-DHB but showed no detectable activity toward VA (Fig. 5, *B* and *C*). In contrast, the Q301P variant exhibited reduced hydroxylation activity toward 3-HBA while acquiring the ability to decarboxylate VA to produce MHQ (Fig. 5, *B* and *C*). Furthermore, the Q301P/W362F double variant displayed a further decrease in 3-HBA hydroxylation activity accompanied by enhanced decarboxylative activity toward VA (Fig. 5, *B* and *C*). Similar trends were observed for reactions with 4-HBA and SA (Fig. S15, *A* and *B*). Notably, introduction of the W362F substitution into the Q301P background shifted the catalytic profile from hydroxylation toward decarboxylative reactions with SA, VA, and 4-HBA, demonstrating that subtle remodeling of the active-site environment can redirect the catalytic outcome (Table 3).

**Table 3:** Kinetic parameters of 3-hydroxybenzoate 6-hydroxylase and its variants

Substrate	Variant	*K*_m_ (μM)	*k*_cat_ (s^−1^)	*k*_cat_/*K*_m_ (s^−1^M^−1^)	Hydroxylation efficiency (%)
3-Hydroxybenzoic acid	Wild type	46 ± 3^a^	35 ± 1^a^	8 × 10^5^^a^	95^a^
	Q301P	11 ± 0.4	0.3 ± 0.01	2.8 × 10^4^	64 ± 0.4
	W362F	108 ± 0.6	7.4 ± 0.1	6.9 × 10^4^	82 ± 1.4
	Q301P/W362F	77 ± 1.1	1.6 ± 0.03	2.1 × 10^4^	30 ± 0.5
4-hydroxybenzoic acid	Wild type	N.D.	N.D.	N.D.	–
	Q301P	50 ± 3.2	0.4 ± 0.01	8.6 × 10^3^	33 ± 1.1
	W362F	N.D.	N.D.	N.D.	–
	Q301P/W362F	58 ± 2.5	1.7 ± 0.04	3.0 × 10^4^	67 ± 10.8
Vanillic acid	Wild type	N.D.	N.D.	N.D.	–
	Q301P	161 ± 5.3	0.2 ± 0.01	1.5 × 10^3^	83 ± 3.6
	W362F	N.D.	N.D.	N.D.	–
	Q301P/W362F	272 ± 6.8	1.5 ± 0.04	5.5 × 10^3^	99 ± 3.4
Syringic acid	Wild type	N.D.	N.D.	N.D.	–
	Q301P	493 ± 25.9	0.4 ± 0.01	9.1 × 10^2^	13 ± 0.4
	W362F	N.D.	N.D.	N.D.	–
	Q301P/W362F	608 ± 4	0.6 ± 0.01	1.0 × 10^3^	31 ± 1.4

Experimental conditions were the same as those described in Table 1. Kinetic analyses of wild-type 3HB6H and its variants were performed using an enzyme concentration of 0.1 μM. Data represent the mean ± SD from three independent experiments.

a Data for 3-hydroxybenzoic acid were obtained from Montersino *et al*. (25).

## Discussion

**Figure 6:**
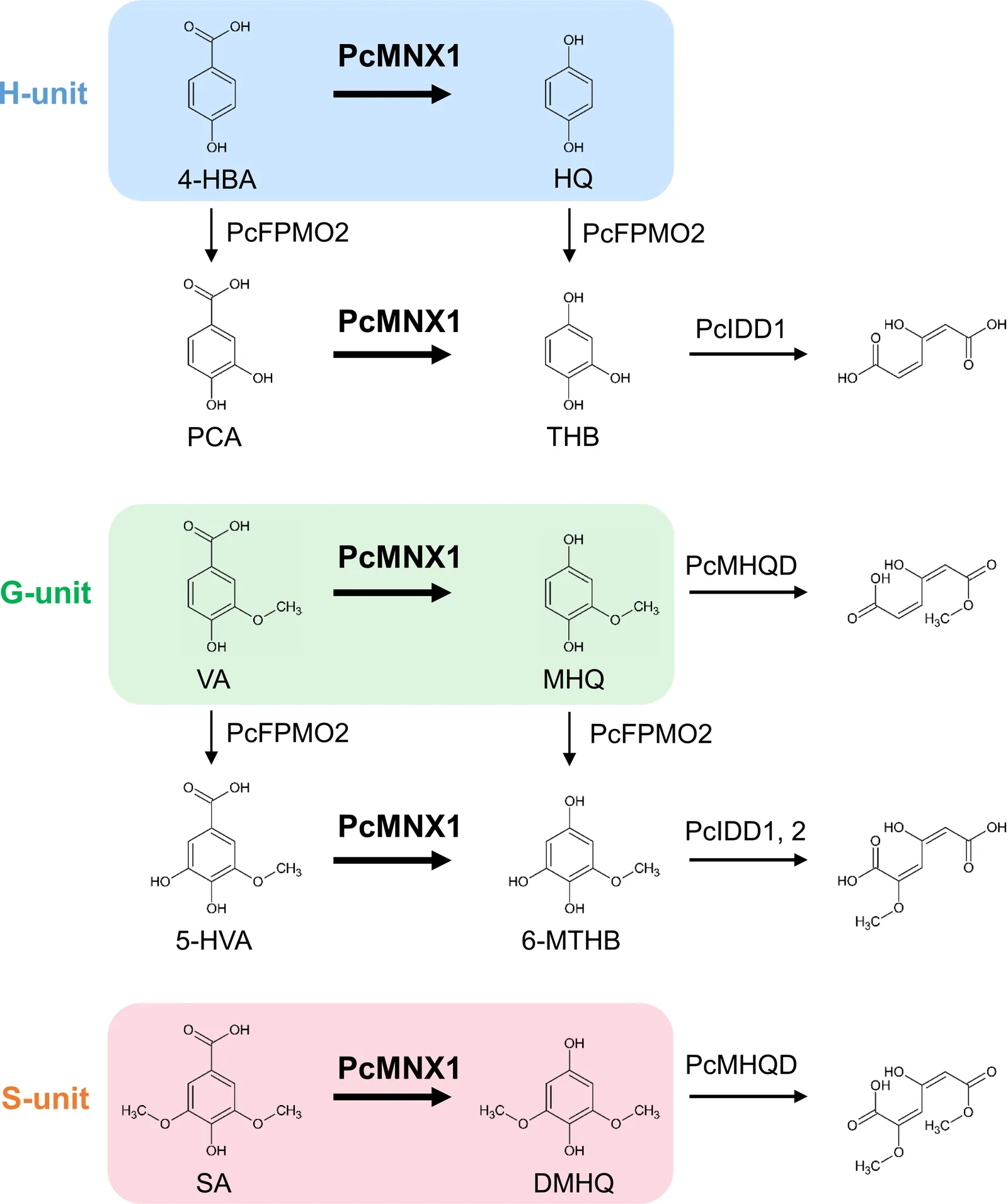
**Proposed metabolic pathways for the conversion of H-, G-, and S-unit lignin-derived aromatics by PcMNX1.***Solid arrows* indicate enzymatically validated reactions catalyzed by PcMNX1, PcFPMO2, PcIDD1, PcIDD2, and PcMHQD. 4-HBA, 4-hydroxybenzoic acid; 5-HVA, 5-hydroxyvanillic acid; 6-MTHB, 6-methoxy-1,2,4-trihydroxybenzene; DMHQ, dimethoxyhydroquinone; HQ, hydroquinone; MHQ, methoxyhydroquinone; PCA, protocatechuic acid; SA, syringic acid; THB, 1,2,4-trihydroxybenzene; VA, vanillic acid.

In this study, we characterized the metabolic conversion of SA by the white-rot fungus *P. chrysosporium* and demonstrated that PcMNX1 catalyzes the oxidative decarboxylation of lignin-derived aromatic compounds, including SA. PcMNX1 converts representatives of H-, G-, and S-type lignin units, including 4-HBA, VA, and SA, indicating that this enzyme likely participates broadly in the metabolism of lignin-derived aromatics in *P. chrysosporium* (Fig. 6). In contrast, GsMNX1 from *G. subvermispora*, despite sharing 69% sequence identity with PcMNX1, exhibits no detectable activity toward SA and shows substantially lower catalytic efficiency toward VA and 4-HBA (10). These findings indicate that even closely related group A FPMOs can display markedly different substrate specificities and suggest that the capacity for S-unit catabolism *via* oxidative decarboxylation may vary considerably among white-rot fungal species.

Metabolic analysis using cell-free extracts indicated that SA catabolism begins with decarboxylative conversion of SA to DMHQ. The enzymatic and structural data obtained in this study demonstrate that PcMNX1 catalyzes this reaction *in vitro*. However, because the genome of *P. chrysosporium* encodes 39 group A FPMOs, it remains possible that one or more of these enzymes possess higher catalytic efficiency toward SA and also contribute to its metabolism *in vivo*. Following this initial step, DMHQ is further metabolized through aromatic ring cleavage catalyzed by PcMHQD. Thus, analogous to previously reported VA degradation pathways (15), SA catabolism in *P. chrysosporium* appears to proceed through a sequential pathway consisting of oxidative decarboxylation catalyzed by PcMNX1, followed by PcMHQD-catalyzed ring cleavage. This pathway contrasts sharply with bacterial mechanisms for SA degradation. In bacteria, SA metabolism typically proceeds *via O*-demethylation reactions mediated by cytochrome P450 systems (such as GcoA/GcoB), the LigM demethylase system, or the VanAB demethylase system, generating catechol-type intermediates prior to aromatic ring cleavage (26, 27, 28, 29). In contrast, the genome of *P. chrysosporium* lacks homologs of the VanAB demethylase system, suggesting that canonical bacterial-type demethylation pathways are absent in this fungus. Although the *P. chrysosporium* genome encodes an extensive repertoire of cytochrome P450 enzymes (approximately 154 genes), none have been experimentally linked to SA *O*-demethylation (30, 31, 32). Given the considerable functional diversification of fungal P450 enzymes (33, 34), it remains possible that some members participate in auxiliary demethylation reactions during aromatic metabolism (12, 13). Nevertheless, the results presented here indicate that oxidative decarboxylation appears to constitute a major entry point for SA degradation in *P. chrysosporium*, defining a fungal-specific metabolic strategy that differs fundamentally from bacterial pathways and relies on decarboxylation rather than *O*-demethylation as the initial activation step (Fig. 6).

The slower rate of SA degradation relative to VA observed *in vivo* (13, 35) is consistent with the kinetic parameters determined for PcMNX1 (Table 1). In particular, the higher *K*_m_ value for SA suggests that PcMNX1 may function as a relatively broad-specificity enzyme capable of acting on multiple lignin-derived aromatic substrates in fungal cells. Such substrate flexibility may be advantageous for white-rot fungi, which encounter complex mixtures of lignin-derived aromatics during wood decomposition. Structural comparison with the closely related enzyme GsMNX1 revealed a key determinant underlying substrate specificity. Beyond the difference in SA activity, PcMNX1 exhibits approximately 48-fold higher catalytic efficiency toward VA than GsMNX1, whereas GsMNX1 displays its highest catalytic efficiency toward PCA (*k*_cat_/*K*_m_ = 2.2 × 10^7^ s^−1^M^−1^) (10). This kinetic disparity, together with the absence of SA activity in GsMNX1, indicates that active-site architecture is a primary determinant of the functional divergence between these two enzymes. In PcMNX1, the residue Ile107 creates sufficient space to accommodate the methoxy substituents of SA, whereas substitution of Leu264 with alanine further increased catalytic efficiency toward this substrate (Fig. S12 and Table 2). These observations indicate that the capacity of PcMNX1 to catalyze SA decarboxylation is largely governed by steric constraints within the substrate-binding cavity. The conservation of these structural determinants among PcMNX1 orthologs in other white-rot fungi further supports this interpretation. Multiple sequence alignment of PcMNX1 orthologs from seven white-rot basidiomycetes—including *T. versicolor, Pycnoporus cinnabarinus, Dichomitus squalens, G. subvermispora, Pleurotus ostreatus, Lentinula novae-zelandiae,* and *Schizophyllum commune*—revealed strong conservation of catalytic and substrate-interacting residues (Fig. S16). Notably, residues corresponding to Ile107 and Leu264 in PcMNX1 were consistently hydrophobic (Ala, Val, Leu, Ile, Met, or Phe) among these orthologs. In *P. cinnabarinus* and *D. squalens*, Ile is conserved at the position corresponding to PcMNX1 Ile107. Moreover, *P. cinnabarinus* possesses an alanine residue at the position corresponding to PcMNX1 Leu264 (Ala300), a substitution predicted to enlarge the active-site cavity and potentially facilitate binding of bulkier substrates such as SA. Together, these observations suggest that PcMNX1-type FPMOs capable of catalyzing the oxidative decarboxylation of SA may be widely distributed among white-rot fungi, contributing to the diversification of lignin-derived aromatic metabolism in this fungal group.

In FPMO-catalyzed monooxygenation of phenolic compounds, the intrinsic *ortho*/*para*-directing effect of the phenolic hydroxyl group increases electron density at the *ortho* and *para* positions of the aromatic ring, thereby disfavoring electrophilic attack at the *meta* position by the flavin C4a-hydroperoxide intermediate (36, 37). In 3HB6H, Gln301 is proposed to constrain substrate orientation within the active-site pocket, promoting hydroxylation of 3-HBA while suppressing decarboxylation (Fig. S14*B*). Although the docking model provides a structural hypothesis for substrate binding, mutational analysis of active-site residues supports this interpretation. Notably, the Q301P/W362F double variant of 3HB6H displayed reduced activity toward 3-HBA while acquiring catalytic activity toward VA, 4-HBA, and SA (Fig. 5*C*; Fig. S15, *A* and *B*; Table 3). These results indicate that the Q301P/W362F substitutions alter substrate positioning within the active-site cavity, thereby shifting the catalytic outcome from aromatic hydroxylation to oxidative decarboxylation.

This functional divergence also reflects differences in the metabolic contexts of bacteria and white-rot fungi. The enzyme 3HB6H was originally identified in the lignin-fragment-degrading bacterium *R. jostii* RHA1, which assimilates VA and SA (38, 39). In this bacterium, VA is converted to PCA by the VanAB *O*-demethylase system, after which PCA undergoes aromatic ring cleavage through the β-ketoadipate pathway. Oxidative decarboxylation of lignin-derived aromatics by FPMOs has not been reported in these bacteria, suggesting that bacterial PcMNX1 homologs primarily function as 3-HBA hydroxylases rather than as decarboxylases of lignin-derived benzoates. In contrast, white-rot fungi predominantly encounter lignin depolymerization products such as VA, SA, and 4-HBA rather than 3-HBA (4, 5, 6, 7). Under these physiological conditions, the ability of PcMNX1 to catalyze the decarboxylative hydroxylation of G-, S-, and H-unit-derived aromatics likely facilitates their subsequent aromatic ring cleavage. These findings illustrate how relatively minor substitutions in active-site residues can redirect catalytic outcomes from hydroxylation to oxidative decarboxylation, highlighting the evolutionary diversification of closely related group A FPMOs in bacterial and fungal aromatic metabolism.

Beyond their metabolic significance, hydroquinone and benzoquinone derivatives are valuable medicinal and industrial compounds used as key intermediates in the synthesis of antitumor agents and other pharmaceuticals (40, 41, 42). Dimethoxybenzoquinone, in particular, functions as an efficient biomimetic electron-transfer mediator in ruthenium-catalyzed aerobic oxidation of alcohols because of the low oxidation potential of its reduced form, DMHQ (43, 44). Currently, these compounds are produced largely through the oxidation of petroleum-derived aromatics using metal-based oxidants, processes that raise environmental and sustainability concerns. In resting cells expressing the PcMNX1_L264A variant, which exhibits enhanced catalytic efficiency toward SA, substantially improved SA conversion was observed (Fig. S13). These findings highlight the potential of PcMNX1 as a promising biocatalyst for the production of value-added hydroquinone derivatives from lignin-derived feedstocks.

Although the docking models and mutagenesis analyses provide structural insights into substrate recognition, substrate-bound crystal structures of PcMNX1 were not obtained. In addition, the mechanistic basis for the contrasting cofactor preferences of PcMNX1 (NADH-preferring) and GsMNX1 (NADPH-preferring) (10) remains to be elucidated. Furthermore, genetic validation will be required to clarify the physiological contribution of PcMNX1 to lignin-derived aromatic metabolism in *P. chrysosporium*.

In summary, we functionally and structurally characterized PcMNX1 (PcFPMO8), a group A FPMO from the basidiomycete *P. chrysosporium*. PcMNX1 catalyzes the oxidative decarboxylation of SA and other lignin-derived aromatic compounds, consistent with a role in facilitating their entry into downstream aromatic ring-cleavage pathways in white-rot fungi. These findings demonstrate how subtle variations in active-site architecture among closely related FPMOs can redirect catalytic outcomes and provide mechanistic insight into the evolutionary diversification of fungal enzymes involved in lignin-derived aromatic metabolism.

## Experimental procedures

### Chemicals and reagents

VA, β-NADH, β-NADPH, riboflavin, DTT, superoxide dismutase (SOD) from bovine erythrocytes and *N,O*-bis(trimethylsilyl)trifluoroacetamide/pyridine (2:1, v/v) were purchased from FUJIFILM Wako Pure Chemical Co., Ltd. 4-HBA, SA, GA, 2-hydroxybenzoic acid, 3-HBA, HQ, MHQ, and 2,5-DHB were obtained from Tokyo Chemical Industry Co., Ltd. PCA was purchased from Nacalai Tesque Inc. 5-HVA was purchased from BLDPharm, Inc. DMHQ, THB, and 6-MTHB were obtained from Apollo Scientific Ltd, Kanto Chemical Co., Inc, and Enamine Ltd, respectively. All chemicals were of analytical grade. Deionized water was prepared using a Milli-Q purification system (Merck Millipore).

### Culture conditions

*P. chrysosporium* (ATCC 34541) was maintained in high-carbon, low-nitrogen (HCLN) medium (pH 4.5) (45, 46, 47) at 37 °C under stationary culture conditions. For experiments, fungal conidia were inoculated into 30 mL of HCLN medium (pH 4.5) (45, 46, 47) in 300-mL Erlenmeyer flasks and incubated at 37 °C under stationary culture conditions. The medium was supplemented with 28 mM d-glucose and 1.2 mM ammonium tartrate as carbon and nitrogen sources, respectively, as described previously (45, 46, 47).

### Cell-free extract preparation and activity analysis

After preincubation of *P. chrysosporium* for 3 days in 30 mL of HCLN medium, SA was added to a final concentration of 1 mM. Following an additional 4 days of incubation, mycelia were harvested by vacuum filtration and stored at −80 °C. For activity analysis, mycelia were ground to a fine powder in liquid nitrogen and resuspended in 20 mM Hepes buffer (pH 7.5) at a concentration of 100 mg mL^−1^. After centrifugation (10,000 × *g*, 4 °C, 10 min), the supernatant was used for enzyme assays. Reaction mixtures contained 2 mM SA, 4 mM NADPH, and 1 mM DTT and were incubated at 20 °C for 3 h. For identification of the conversion products, reaction mixtures were extracted twice with ethyl acetate at pH 2.0, evaporated to dryness, and derivatized with *N,O*-bis(trimethylsilyl)trifluoroacetamide/pyridine (2:1, v/v). The TMS derivatives of the conversion products were analyzed by GC–MS.

### Construction of the gene expression system

The full-length *P. chrysosporium PcFPMO8* gene was PCR-amplified using KOD One DNA polymerase (Toyobo), the primer combinations specified in Table S3, and a DNA Thermal Cycler 2400 (Takara Bio, Otsu, Japan) under the following conditions: 25 cycles of denaturation at 98 °C for 10 s, annealing at 56 °C for 10 s, and extension at 68 °C for 10 s. PCR products were separated by 1.0% agarose gel electrophoresis. Gels were stained with ethidium bromide and visualized using a Molecular Imager FX system (Bio-Rad). Amplified DNA fragments were purified using a FastGene Gel/PCR Extraction Kit (Nippon Genetics Co., Ltd) and ligated into a BamHI-digested pQE60 vector (Invitrogen) using NEBuilder HiFi DNA Assembly Master Mix (New England Biolabs). The full-length *3HB6H* gene, codon-optimized for heterologous expression in *E*. *coli* (Text S1), was synthesized by Eurofins Genomics and inserted into the pBAD/TOPO vector (Invitrogen). The recombinant plasmids harboring *PcFPMO8* and *3HB6H* were transformed into *E. coli* JM109 (Invitrogen) and TOP10 (Invitrogen), respectively, and transformants were selected on lysogeny broth agar plates containing ampicillin. The identity of the recombinant plasmids was confirmed by DNA sequencing. Plasmids for recombinant PcFPMO4–PcFPMO7 production were constructed using the same strategy. PcMNX1 and 3HB6H variants were generated by site-directed mutagenesis using inverse PCR with the primers listed in Table S3, followed by DpnI digestion (Takara).

### Heterologous expression and purification of PcFPMOs

*E. coli* JM109 cells harboring each *PcFPMO* expression vector were grown at 37 °C with constant shaking in lysogeny broth medium supplemented with 100 μg mL^−1^ ampicillin and 50 μg mL^−1^ riboflavin until the OD600 reached 0.6. Protein expression was induced by the addition of 100 μM IPTG, and the cultures were incubated for an additional 6 h at 37 °C. Cells were harvested by centrifugation (3000 × *g*, 25 °C, 10 min), and the cell pellet was resuspended in buffer A (20 mM Hepes, pH 7.5, 10% (w/v) glycerol, and 1 mM phenylmethylsulfonyl fluoride). Cells were lysed by sonication (5 × 30 s) using a Q700 sonicator (Qsonica). Insoluble material was removed by centrifugation (10,000 × *g*, 4 °C, 10 min), and the supernatant was retained for protein purification. Expression of 3HB6H and its variants was performed as described previously (18).

The crude extract was loaded onto a nickel-affinity chromatography column (Cytiva) equilibrated with buffer B (buffer A containing 200 mM NaCl). The column was washed with buffer B, and bound proteins were eluted using a linear gradient of 0 to 0.3 M imidazole in buffer B. Fractions exhibiting a yellow color, indicative of FAD binding, were pooled and further purified by size-exclusion chromatography on a Superdex 200 HR 10/30 column (Cytiva) equilibrated with buffer B. The purified proteins were collected, and their absorption spectra were recorded using a SpectraMax spectrophotometer (Molecular Devices).

### FPMO enzyme assay

FPMO activity was determined and reaction products were identified as described previously (45, 46, 47), with slight modifications. Reaction mixtures (0.5 mL) contained 0.1 μM FPMO, 2 mM NAD(P)H, 1 mM substrate (from a 100 mM stock solution in dimethyl sulfoxide (DMSO)), 0.2 mM DTT, and 6 μg mL^−1^ SOD in 20 mM Hepes buffer (pH 8.0). DTT was included to reduce quinones generated by auto-oxidation to the corresponding hydroquinones, whereas SOD was included to scavenge superoxide produced during hydroquinone auto-oxidation, thereby stabilizing the hydroquinone products throughout the assay (Fig. S17). Reactions were initiated by the addition of NAD(P)H and incubated at 20 °C for 1 h. Reactions were terminated by adding 10 μL of 6 N HCl. Reaction products were identified by GC–MS as described previously (32, 46, 47).

Kinetic parameters were determined under the following conditions. Reaction mixtures (0.5 mL) contained enzyme, NAD(P)H, and 5 μL of substrate solution (0–500 mM in DMSO) in 20 mM Hepes buffer (pH 8.0). Unless otherwise indicated, NAD(P)H was used at a concentration of 200 μM. For determination of the kinetic parameters for NAD(P)H, the cofactor concentration was varied over the indicated range. For PcMNX1, an enzyme concentration of 5 nM was used when NAD(P)H, 4-HBA, VA, or PCA was varied, whereas 0.1 μM enzyme was used when SA, GA, 5-HVA, or 3-HBA was varied. The PcMNX1 I107M and L264A variants were analyzed using enzyme concentrations of 0.1 μM and 20 nM, respectively, whereas WT 3HB6H and its variants were analyzed using an enzyme concentration of 0.1 μM. Reactions were initiated by the addition of NAD(P)H at 20 °C, and the decrease in absorbance at 340 nm was monitored using a SpectraMax spectrophotometer (Molecular Devices) equipped with a 1-cm-path-length quartz cuvette. Initial rates were calculated from the change in absorbance during the first 10 s of the reaction. Background rates of NAD(P)H consumption measured in reaction mixtures lacking substrate (1.5 ± 0.2 μM min^−1^ for NADH and 1.3 ± 0.1 μM min^−1^ for NADPH) were subtracted from the corresponding rates measured in the presence of substrate. The kinetic parameters *K*_m_ and *k*_cat_ were obtained by fitting the initial rate data to the Michaelis–Menten equation using Origin version 6.0 (OriginLab).

Hydroxylation efficiency was determined under the same conditions used for kinetic analyses and was calculated as the ratio of substrate hydroxylation to NAD(P)H oxidation. NAD(P)H consumption was monitored spectrophotometrically at 340 nm, whereas substrate hydroxylation was quantified by HPLC.

### Analytical methods

GC–MS analysis was performed using a GCMS-QP2010 system (Shimadzu) operated at an electron ionization energy of 70 eV and equipped with a 30-m fused-silica capillary column (DB-5, J&W Scientific). The oven temperature was programmed from 80 °C to 320 °C at a rate of 8 °C min^−1^ and the injector temperature was maintained at 280 °C. Reaction products were identified by comparing their GC retention times and mass fragmentation patterns with those of authentic standards (46, 47, 48). Compounds for which authentic standards were unavailable were identified by comparison of their mass spectra with entries in the National Institute of Standards and Technology Mass Spectral Library.

HPLC analysis was performed using a Nexera XR system (Shimadzu) equipped with a Shim-pack GIST C18-AQ column (2.1 × 100 mm; Shimadzu). The mobile phase consisted of a 5% acetonitrile in 0.1% phosphoric acid for the first 3 min, followed by a linear gradient to 100% acetonitrile over 15 min at a flow rate of 0.2 mL min^−1^. Eluted compounds were monitored by UV absorbance at 254 and 280 nm.

### Crystallization of PcMNX1

PcMNX1 was crystallized using the sitting-drop vapor-diffusion method. Drops containing 0.75 μL of PcMNX1 solution (12 mg mL^−1^) and 0.75 μL of reservoir solution (0.2 M calcium acetate hydrate, 0.1 M sodium cacodylate trihydrate, pH 6.5, and 18% (w/v) polyethylene glycol 8000) were equilibrated against the reservoir solution at 4 °C. Crystals appeared after 3 days. Before data collection, crystals were briefly transferred to reservoir solution supplemented with 10% (w/v) glycerol as a cryoprotectant and flash-cooled in liquid nitrogen.

### Data collection and structure determination

Diffraction data were collected at beamline BL-17A of the Photon Factory, High Energy Accelerator Research Organization (KEK), using a PILATUS3 S 6M detector (DECTRIS). Crystals were maintained at −178 °C in a nitrogen cryostream during data collection.

Diffraction images were processed with XDS (implemented in the PREMO data-processing system at the Photon Factory), and scaled with AIMLESS in the CCP4i program suite. The structure of PcMNX1 was solved by molecular replacement using MOLREP within CCP4i and refined with REFMAC5 (49). Data collection and refinement statistics are summarized in Table S1. Atomic coordinates and structure factors have been deposited in the Protein Data Bank (PDB) under accession code 8X38. Structural figures were prepared using PyMOL (PyMOL Molecular Graphics System, version 2.5.0; Schrödinger, LLC).

### Structural analysis of PcMNX1, GsMNX1, and 3HB6H

Structural models of PcMNX1 and PcFPMO4–PcFPMO7 in the catalytically relevant FAD “*in*” conformation, together with variants of 3HB6H, were generated using AlphaFold3 (50) with default parameters in the ColabFold environment. Ligand docking simulations were performed using the Molecular Operating Environment (Chemical Computing Group Inc.). Docked complexes were visualized and analyzed using PyMOL. Crystal structures of GsMNX1 (PDB ID: 8R2T), 3HB6H (PDB ID: 4BJZ), and the 3HB6H H213S variant complexed with 3-HBA (PDB ID: 4BK1) were obtained from the PDB.

### SA bioconversion by resting cells

For resting-cell bioconversion assays, enzyme production was carried out as described in the “*Heterologous expression and purification of PcFPMOs*” section. Cells were harvested by centrifugation (3000 × *g*, 25 °C, 10 min), washed twice with 20 mM Hepes buffer (pH 7.5) containing 50 mM NaCl, and resuspended in the same buffer to an OD600 of 20. Bioconversion reactions were performed in a total volume of 5 mL at 20 °C with shaking at 100 rpm. Reactions were initiated by the addition of SA to a final concentration of 0.5 mM from a 100 mM stock solution prepared in DMSO, together with 0.1% (w/v) glucose. During the reaction, 500-μL aliquots were withdrawn and centrifuged (10,000 × *g*, 4 °C, 10 min). The resulting supernatants were filtered before HPLC analysis.

## Data availability

All data generated or analyzed during this study are included in this article and its supporting information.

## Supporting information

This article contains supporting information.

## Conflict of interest

The authors declare that they have no conflicts of interest with the contents of this article.
