# Supplementary material for: Structural basis for oxidative decarboxylation of lignin-derived aromatics by the fungal flavoprotein monooxygenase PcMNX1
Source: J Biol Chem. 2026 Aug 7;302(10):113411. doi: 10.1016/j.jbc.2026.113411 (PMC13559804; doi:10.1016/j.jbc.2026.113411)
Supplement: Supplementary file 1 [file mmc1.docx]

**Supporting Information for**

**Structural basis for oxidative decarboxylation of lignin-derived aromatics by the fungal flavoprotein monooxygenase PcMNX1**

Reini Mori^1^, Hiromitsu Suzuki^1^, Takuya Ishida^2^, Kiyota Sakai^1^, Sora Yamaguchi^2^, Naoki Sunagawa^2^, Kiyohiko Igarashi^2^, Masashi Kato^1^, Motoyuki Shimizu^1#^

Running Head: PcMNX1 decarboxylates lignin-derived aromatics

**^#^To whom correspondence should be addressed:** Motoyuki Shimizu, moshimi@meijo-u.ac.jp.

**This document contains the following:**

Supplemental figures: Fig. S1–S17

Supplemental tables: Tables S1–S3

Supplemental text: Text S1

**Figure S1. Phylogenetic analysis of flavoprotein monooxygenases (FPMOs) from *Phanerochaete chrysosporium*.**

(A) Bootstrap consensus phylogenetic tree of 57 FPMO protein sequences identified in the *P. chrysosporium* genome (<https://mycocosm.jgi.doe.gov/Phchr4_2/Phchr4_2.home.html>). Thirty-nine sequences were classified as group A FPMOs and 18 as group B FPMOs. Numbers correspond to the JGI MycoCosm protein IDs. (B) Bootstrap consensus phylogenetic tree of 39 *P. chrysosporium* FPMOs together with 27 biochemically characterized group A FPMOs. The clade of group A FPMOs predicted to catalyze *para*-hydroxylation is highlighted. The identifiers for GsMNX1 and TvMNX1 correspond to the JGI MycoCosm protein IDs (<https://mycocosm.jgi.doe.gov/Cersu1/Cersu1.home.html> and https://mycocosm.jgi.doe.gov/trave1/trave1.home.html), whereas the identifiers for the other characterized FPMOs correspond to UniProt accession numbers.

**Figure S2. Multiple sequence alignment of PcFPMO4****–8 and biochemically characterized group A FPMOs.**

The alignment includes PcFPMO4–8 from *P. chrysosporium* (JGI MycoCosm protein IDs: 6222556, 6342839, 6212972, 6212917 and 6247437), GsMNX1 from *Gelatoporia subvermispora* (JGI MycoCosm protein ID: 120062), CpMNX1 from *Candida parapsilosis* CBS604 (UniProt accession number: G8B709), and 3-hydroxybenzoate 6-hydroxylase (3HB6H) from *Rhodococcus jostii* RHA1 (UniProt accession number: Q0SFK6), all belonging to the same clade shown in Fig. S1B. Completely conserved residues are indicated in black. FAD-binding motifs (fingerprints) are highlighted in yellow, substrate-interacting residues in blue, and catalytic residues in magenta.

**Figure S3. SDS–PAGE analysis and UV–visible absorption spectra of recombinant PcFPMOs.**

(A) SDS–PAGE analysis of purified recombinant PcFPMO5, PcFPMO7, and PcFPMO8. Lane M, molecular-mass markers. (B) UV–visible absorption spectra of purified PcFPMO5, PcFPMO7, and PcFPMO8.

**Figure S4. Total ion chromatograms of PcFPMO5 and PcFPMO7 reactions.**

(A–C) Reaction mixtures containing SA (A), 4-HBA (B), or VA (C) were incubated with PcFPMO5 or PcFPMO7 and analyzed by GC–MS after TMS derivatization. Experiments were performed in triplicate, and representative chromatograms are shown.

**Figure S5. GC–MS analysis of reaction products generated by PcMNX1.**

(A–D) Reaction products generated from PCA (A), GA (B), 5-HVA (C), and 3-hydroxybenzoic acid (3-HBA) (D) were analyzed by GC–MS after TMS derivatization. Mass spectra of the reaction products—THB (A), TetraHB (B), MTHB (C), and 2,5-DHB (D)—were obtained from GC peaks with retention times of 30.8 min (A), 36.0 min (B), 35.0 min (C), and 36.5 min (D). Experiments were performed in triplicate, and representative chromatograms are shown.

**Figure S6. Effects of pH and temperature on PcMNX1 activity.**

(A) pH dependence of PcMNX1 activity toward VA over the pH range of 3.0–10.0. Reactions were performed in 20 mM sodium acetate buffer (pH 3.0–6.0; ●), 20 mM HEPES–NaOH buffer (pH 6.0–8.0; ■), and 20 mM Tris–HCl buffer (pH 8.0–10.0; ▲). (B) Temperature dependence of PcMNX1 activity measured between 10 °C and 70 °C.

**Figure S7. FAD-binding site of PcMNX1.**

(A) Close-up view of the interactions between PcMNX1 and the bound FAD cofactor (PDB ID: 8X38). Carbon atoms of PcMNX1 and FAD are shown in gray and yellow, respectively, whereas nitrogen, oxygen, and phosphorus atoms are shown in blue, red, and orange, respectively. Hydrogen-bond interactions between PcMNX1 and FAD are indicated by yellow dashed lines. Water molecules are shown as red spheres. (B) Close-up view showing stabilization of the FAD isoalloxazine ring through π–π stacking interactions with Trp274.

**Figure S8. Structural models of PcMNX1 with FAD in the *“in”* conformation predicted by AlphaFold3.**

(A) Structural model of PcMNX1 with FAD in the catalytically relevant *“in”* conformation predicted by AlphaFold3. (B) Structural superposition of the PcMNX1 model and GsMNX1 with FAD bound in the *“in”* conformation (PDB ID: 8R2T). Carbon atoms of FAD in PcMNX1 and GsMNX1 are shown in yellow and magenta, respectively.

**Figure S9. Proposed mechanism for the decarboxylative hydroxylation catalyzed by PcMNX1.**

The reaction is initiated by electrophilic attack of the flavin C4a-hydroperoxide intermediate on the aromatic ring of vanillic acid. His247 is proposed to facilitate proton transfer during catalysis, thereby stabilizing the reaction intermediate. Subsequent rearrangement and decarboxylation yield methoxyhydroquinone while regenerating the oxidized flavin cofactor. Curved arrows indicate the proposed electron flow during the reaction.

**Figure S10. Structural models of PcFPMO4–7.**

(A–D) AlphaFold3-predicted structural models of PcFPMO4–7. Superposition of the active-site pocket of PcMNX1 docked with vanillic acid and those of PcFPMO4 (A), PcFPMO5 (B), PcFPMO6 (C), and PcFPMO7 (D). Residues corresponding to Gln79, Tyr134, His247, and Tyr251 in PcMNX1 are shown in gray in all panels.

**Figure S11. Structural comparison of PcMNX1 and GsMNX1 and functional analysis of the PcMNX1_I107M variant.**

(A, B) Putative ligand-bound structures of PcMNX1 (A) and GsMNX1 (PDB ID: 8R2T) (B) generated by docking SA into the active site using the MOE program. Carbon atoms of PcMNX1, GsMNX1, and SA are shown in gray, magenta, and orange, respectively. The mesh representation illustrates the shape and volume of the active-site cavity. (C, D) GC–MS analysis of reaction mixtures containing SA (C) and VA (D) after TMS derivatization. The products dimethoxyhydroquinone (DMHQ) (C) and methoxyhydroquinone (MHQ) (D) were detected at retention times of 33.4 min and 28.7 min, respectively. Chromatograms corresponding to reactions without enzyme, wild-type PcMNX1, and the PcMNX1_I107M variant are shown in black, red, and purple, respectively. Experiments were performed in triplicate, and representative chromatograms are shown.

**Figure S12. Structural and functional characterization of the PcMNX1_L264A variant.** (A, B) Putative ligand-bound structures of PcMNX1 (A) and the PcMNX1_L264A variant (B) generated using AlphaFold3. SA was docked into the active site using the MOE program. Carbon atoms of PcMNX1 and SA are shown in gray and orange, respectively. The mesh representation illustrates the shape and volume of the active-site cavity. (C, D) GC–MS analysis of reaction mixtures containing SA (C) and VA (D) after TMS derivatization. The products DMHQ (C) and MHQ (D) were detected at retention times of 33.4 min and 28.7 min, respectively. Chromatograms corresponding to reactions without enzyme, wild-type PcMNX1, and the PcMNX1_L264A variant are shown in black, red, and pink, respectively. Experiments were performed in triplicate, and representative chromatograms are shown.

**Figure S13. Bioconversion of syringic acid by resting cells.**

(A) SDS–PAGE analysis of soluble proteins extracted from resting *Escherichia coli* cells expressing the empty pQE60 vector, wild-type PcMNX1, or the PcMNX1_L264A variant. Equal amounts of cells were used for protein extraction. Lane M, molecular-mass markers. (B) Time course of SA conversion by resting cells. Resting-cell bioconversion assays were conducted at an optical density at 600 nm (OD_600_) of 20 in 20 mM HEPES buffer (pH 7.5) containing 50 mM NaCl, 0.5 mM SA, and 0.1% (w/v) glucose at 20 °C with shaking at 100 rpm. SA and DMHQ concentrations were quantified by HPLC. Data represent the mean ± standard deviation from three independent experiments.

**Figure S14. Structural comparison of PcMNX1 and 3-hydroxybenzoate 6-hydroxylase (3HB6H).**

(A, B) Putative ligand-bound structures generated by docking VA or 3-HBA into the active sites of PcMNX1 (A) and 3HB6H (PDB ID: 4BK1) (B). Because the latter structure was solved as the H213S mutant, PDB ID: 4BJZ was used to visualize His213. Carbon atoms of PcMNX1, 3HB6H, VA and 3-HBA are shown in gray, orange, green and cyan, respectively. The mesh illustrates the shape and volume of the active-site pocket.

**Figure S15. Functional characterization of 3HB6H variants.**

(A, B) GC–MS analysis of reaction products generated from 4-HBA (A) and SA (B) after TMS derivatization. The products hydroquinone (HQ) (A) and DMHQ (B) were detected at retention times of 23.9 min and 33.4 min, respectively. Experiments were performed in triplicate, and representative chromatograms are shown.

**Figure S16. Multiple sequence alignment of PcMNX1 orthologs from white-rot basidiomycetes.** The alignment includes PcMNX1 orthologs from *Gelatoporia subvermispora,* *Trametes versicolor,* *Pycnoporus cinnabarinus*, *Pleurotus ostreatus*, *Lentinula* novae-zelandiae, *Schizophyllum commune,* and *Dichomitus squalens*. Accession numbers and JGI MycoCosm protein IDs are provided in Table S2. Completely conserved residues are highlighted in black. Conserved FAD-binding motifs (fingerprints), substrate-interacting residues, catalytic residues, and residues implicated in active-site cavity formation are highlighted in yellow, blue, magenta, and green, respectively.

**Figure S17. Stability of VA and MHQ under the enzyme assay conditions.** Representative GC chromatograms (A) and photographs (B) of the corresponding reaction mixtures are shown. VA and MHQ (1 mM) were analyzed immediately after preparation (0 h) or after incubation for 1 h under the standard assay conditions in the absence of additives, in the presence of DTT alone, or in the presence of both DTT and SOD. DTT and SOD markedly improved the stability of MHQ, whereas the stability of VA was unaffected.

**Table S1. Crystallographic data collection and refinement statistics for PcMNX1.**

| **Parameter** | **PcMNX1** |
| --- | --- |
| Space group | *P*2_1_2_1_2_1_ |
| Unit cell parameters (Å) | a = 45.63, b = 97.13, c = 106.60 |
| Resolution (Å) | 48.56–1.67 (1.70–1.67) |
| Observed reflections | 732950 |
| Unique reflections | 55920 |
| Redundancy | 13.1 (12.7) |
| Completeness (%) | 100.0 (100.0) |
| R_merge_ | 0.056 (1.025) |
| CC_1/2_ | 1.000 (0.869) |
| *<I/σ(I)>* | 28.8 (2.7) |
| *R*_work_ | 0.179 |
| *R*_free_ | 0.214 |
|  |  |
| **B-factors (Å^2^)** |  |
| Protein | 29.87 |
| Solvent | 29.63 |
| Ligand (FAD) | 23.98 |
| Ligand (acetate [ACT]) | 38.56 |
|  |  |
| **Ramachandran statistics** |  |
| Allowed (%) | 95.54 |
| Generously allowed (%) | 3.99 |
| Disallowed (%) | 0.47 |
|  |  |
| **RMSD** |  |
| Bonds (Å) | 0.01 |
| Angles (°) | 1.64 |
| PDB ID | 8X38 |

Values in parentheses are for the highest-resolution shell.

**Table S2. Accession numbers and JGI MycoCosm protein IDs of PcMNX1 orthologs from white-rot basidiomycetes.**

| **White-rot basidiomycete** | **Database** | **ID/Accession number** |
| --- | --- | --- |
| *Gelatoporia subvermispora* | JGI MycoCosm (Protein ID) | 120062 |
| *Trametes versicolor* |  | 175239 |
| *Pycnoporus cinnabarinus* |  | 1011519 |
| *Dichomitus squalens* |  | 113156 |
| *Pleurotus ostreatus* | NCBI (Accession No.) | XP_036630341.1 |
| *Lentinula novae-zelandiae* |  | KAJ3868811.1 |
| *Schizophyllum commune* |  | KAI4521224.1 |

**Table S3. Oligonucleotide primers used for recombinant protein expression.**

Gene-specific primers were designed based on genomic sequence data available from the DOE Joint Genome Institute (*Phanerochaete chrysosporium* database; https://mycocosm.jgi.doe.gov/Phchr4_2/Phchr4_2.home.html). Primers for the 3-hydroxybenzoate 6-hydroxylase (3HB6H) variants were designed using the codon-optimized sequence (Text S1) for heterologous expression in *Escherichia coli*.

| **Primer** | **Protein ID** | **Nucleotide sequence (5′–3′)** |
| --- | --- | --- |
| **Cloning for recombinant protein production** |  | |
| PcFPMO4-f | 6222556 | AGGAGAAATTAACCATGGGAGGATCCCAGGAGCGACCTATACAAGC |
| PcFPMO4-r |  | TGGTGATGGTGATGAGATCTGGATCCGAGCACCTCGATGCTCTGCG |
| PcFPMO5-f | 6342839 | AGGAGAAATTAACCATGGGAGGATCCTCCTCAGGCGAGTCAAGCAC |
| PcFPMO5-r |  | TGGTGATGGTGATGAGATCTGGATCCACTGACCTCCGCCTCGATCA |
| PcFPMO6-f | 6212972 | AGGAGAAATTAACCATGGGAGGATCCGCTTCCTCCAAGGTCGCGTC |
| PcFPMO6-r |  | TGGTGATGGTGATGAGATCTGGATCCTTGCGCCGAGATGGACAGGG |
| PcFPMO7-f | 6212917 | AGGAGAAATTAACCATGGGAGGATCCCCGACCGCTGTACACTCTGG |
| PcFPMO7-r |  | TGGTGATGGTGATGAGATCTGGATCCAGCGTCGTTACGTGATAGAG |
| PcFPMO8-f | 6247437 | AGGAGAAATTAACCATGGGAGGATCCTCCTCTGAGCCGATCACTGC |
| PcFPMO8-r |  | TGGTGATGGTGATGAGATCTGGATCCGAGGCGGCCGTTGACCTTGA |
| **Site-directed mutagenesis** |  | |
| PcMNX1_Q79A-f | 6247437 | GGGCATCGCGGTCACCCCCAACG |
| PcMNX1_Q79A-r |  | CGTTGGGGGTGACCGCGATGCCC |
| PcMNX1_Y134A-f |  | GAGTACGGCGCGCCCGCCTACCACATACACCGC |
| PcMNX1_Y134A-r |  | CTCCATCTTATCGCCCCACTTGGTATAGCC |
| PcMNX1_H247A-f |  | ATGGGCCCTGGCACGGCTTTGATGGCATACAAC |
| PcMNX1_H247A-r |  | CCACGCTGTCATCTCCGGGGTATCGACGAA |
| PcMNX1_Y251A-f |  | CACGCATTTGATGGCAGCCAACATTCGTGCGAAG |
| PcMNX1_Y251A-r |  | CTTCGCACGAATGTTGGCTGCCATCAAATGCGTG |
| PcMNX1_I107M-f |  | CCCGAGGCGATGGTCTTCCGGCGGTAC |
| PcMNX1_I107M-r |  | GTACCGCCGGAAGACCATCGCCTCGGG |
| PcMNX1_L264A-f |  | CAACATGGTGCTGGCGCATCCGGATGACGG |
| PcMNX1_L264A-r |  | CCGTCATCCGGATGCGCCAGCACCATGTTG |
| 3HB6H_Q301P-f | UniProt ID  Q0SFK6 | GCACATCCGCCGCTGCCGTATCTGGCCAG |
| 3HB6H_Q301P-r |  | CGCATCACCCAGCAGAATCATGCGGCCATC |
| 3HB6H_W362F-f |  | ATGTGGGGCGAACTGTTCCACCTGGATGGCACC |
| 3HB6H_W362F-r |  | GGCCGGTGGTCAGAATGCGGTTGCAGCGC |

f, forward primer; r, reverse primer.

Underlined nucleotides indicate the substitutions introduced to generate the designated amino acid replacements.

**Text S1.** Codon-optimized nucleotide sequence encoding 3-hydroxybenzoate 6-hydroxylase (3HB6H) for heterologous expression in *Escherichia coli*.

AGCAACCTGCAGGATGCGCGTATTATTATTGCGGGTGGCGGCATTGGCGGCGCGGCGAATGCGCTGGCCCTGGCGCAGAAAGGTGCCAATGTGACCCTGTTTGAACGTGCGAGCGAATTCGGCGAAGTGGGCGCGGGCCTGCAGGTGGGCCCGCACGGCGCGCGTATTCTGGATAGTTGGGGTGTTCTGGATGATGTTCTGAGCCGTGCATTTCTGCCGAAAAACATTGTGTTCCGCGATGCGATTACCGCGGAAGTGCTGACCAAAATTGATCTGGGCAGCGAATTTCGCGGCCGCTACGGCGGCCCGTATTTTGTGACGCACCGCAGCGATCTGCATGCGACCCTGGTGGATGCCGCCCGCGCAGCGGGCGCGGAACTGCACACCGGGGTGACCGTGACCGATGTGATTACCGAAGGCGACAAAGCGATTGTGTCAACCGATGATGGCCGCACCCACGAAGCAGATATTGCCCTGGGTATGGATGGCCTGAAAAGTCGCCTGCGCGAAAAAATTAGCGGCGATGAACCGGTCTCATCGGGCTATGCGGCCTACCGCGGCACCACCCCGTATCGCGATGTGGAACTGGATGAAGATATTGAAGATGTGGTTGGCTACATCGGTCCGCGCTGCCATTTTATCCAGTATCCGCTGCGCGGTGGCGAAATGCTGAACCAGGTGGCCGTGTTTGAAAGCCCGGGCTTCAAAAACGGCATTGAAAACTGGGGCGGCCCGGAAGAACTGGAACAGGCCTATGCGCACTGCCATGAAAACGTGCGCCGCGGCATTGATTATCTGTGGAAAGATCGCTGGTGGCCGATGTATGATCGCGAACCGATTGAAAATTGGGTGGATGGCCGCATGATTCTGCTGGGTGATGCGGCACATCCGCCGCTGCAGTATCTGGCCAGCGGCGCGGTGATGGCGATTGAAGATGCAAAATGCCTGGCGGATTATGCGGCCGAAGATTTTAGCACCGGTGGCAATAGCGCGTGGCCGCAGATTCTGAAAGAAGTGAACACCGAACGCGCCCCGCGCTGCAACCGCATTCTGACCACCGGCCGTATGTGGGGCGAACTGTGGCACCTGGATGGCACCGCCCGCATTGCGCGTAACGAATTATTTCGTACCCGCGATACTAGCAGCTATAAATATACCGATTGGCTGTGGGGCTACAGCAGCGATCGCGCCAGC
